# Proposed Mechanism for the High-Yield Polymerization of Oxyethyl Propiolates with Rh Complex Catalyst Using the Density Functional Theory Method

**DOI:** 10.3390/polym11010093

**Published:** 2019-01-08

**Authors:** Yoshiaki Yoshida, Yasuteru Mawatari, Masayoshi Tabata

**Affiliations:** 1 Graduate School of Engineering, Muroran Institute of Technology, 27-1 Mizumoto-cho, Muroran, Hokkaido 050-8585, Japan; yyoshida@moleng.fuk.kindai.ac.jp; 2 Research Center for Environmentally Friendly Materials Muroran Institute of Technology, 27-1 Mizumoto-cho, Muroran, Hokkaido 050-8585, Japan; 3 Center of Environmental Science and Disaster Mitigation for Advanced Research, Muroran Institute of Technology, 27-1 Mizumoto-cho, Muroran, Hokkaido 050-8585, Japan; 4 Faculty of Science and Technology, Department of Applied Chemistry and Bioscience, Chitose Institute of Science and Technology, Bibi 65-758, Hokkaido Chitose 066-8655, Japan

**Keywords:** mono-substituted acetylene, Rh complex catalyst, propiolic ester, oxyethylene group

## Abstract

In this study, poly(oxyethyl propiolate)s (POP)s featuring various oxyethylene derivatives are synthesized using a [Rh(norbornadiene)Cl]_2_ catalyst. In particular, POPs featuring the normal oxyethylene chain in the side-chain exhibit excellent yields and high molecular weights in methanol and *N*,*N*-dimethylformamide at 40 °C, compared with poly(*n*-alkyl propiolate)s (P*n*AP)s. The high reactivity of the oxyethyl propiolate (OP) monomers is clarified by considering the time dependences of the polymerization yields of OPs and alkyl propiolates (Aps). Furthermore, the monomer structure and intermediate conformation of the Rh complex are optimized using Density Function theory (DFT) methods (B3LYP/6-31G** and B3LYP/LANL2DZ) and a polymerization mechanism is proposed.

## 1. Introduction

Recently, mono-substituted polyacetylenes (SPAs), which are attractive helical and π-conjugated polymers, have been applied in stimuli-responsive materials, molecular recognition, gas permeability, and optical resolution [1,2,3,4,5,6,7,8,9]. During the past three decades, many SPA derivatives have been synthesized, featuring a variety of functional groups in their side-chains. They have been synthesized using various metal catalysts, such as the Ziegler–Natta catalyst, the Luttinger catalyst, the Wilkinson catalyst, metathesis catalysts, and Rh-based catalysts [10,11,12,13,14,15,16,17,18,19]. In particular, the Rh bidentate catalyst, [Rh(nbd)Cl]_2_ (nbd = norbornadiene), has become an important catalyst for the synthesis of helical SPAs with *cis–transoid* geometrical structures from various mono-substituted acetylene (SA) monomers. This polymerization proceeds through not a metathesis mechanism but a coordination–insertion mechanism [13,14,15,16,17,18,19]. Moreover, an active monodentate Rh complex catalyst can be generated in situ from a Rh bidentate catalyst when amine, alcohol, or even water is used as the cocatalyst or solvent [13,14,15,16,17,18,19,20,21,22]. The [Rh(nbd)Cl]_2_/amine binary catalyst system has been reported to be a significantly effective catalyst for the polymerization of various phenyl acetylene derivatives and propargyl esters [23,24,25,26,27,28,29,30,31,32,33,34]. Propiolic esters, however, are sluggish when used as a monomer for the Rh catalyst system, due to their strong acidity. In fact, poly(alkyl proiolate)s (PAP)s exhibiting various alkyl chains have been obtained at low yields in alcohol solvents [20,21,22,35,36,37,38,39,40,41]. Masuda et. al. reported a synthesis of PAP with the Rh catalysts of not only dimer complex containing cyclic dienes but also of cationic and zwitterionic complex [22]. They also concluded that propiolic esters had low reactivity toward polymerization with Rh catalyst due to the electron-withdrawing effect of the ester group. In addition, we clarified that PAPs except methyl propiolate were never polymerized by the metathesis catalysts such as Molybdenum(III) chloride (MoCl_3_) and tungsten(VI) chloride (WCl_6_) [35]. On the other hand, pentynoic acid derivatives, which have ethylene spacer between triple bond and ester moieties (HC≡CCH_2_CH_2_COOR), were polymerized by the binary catalyst system with Rh complex and amine because of their fairly weak acidity compared to that of PAPs [36]. It has recently been shown that PAPs can have both a helical contracted *cis–cisoid* structure and a stretched *cis–transoid* structure in solution. Furthermore, these helical polymers have been shown to exhibit a unique accordion-like helix oscillation (HELIOS) [42,43,44]. The high yield preparation of PAPs is therefore important, though challenging, the self-motion exhibited by their molecules could probably be applied as a molecular motor, and as a switching material such as a biomolecule. This study details an attempt to find reactive AP monomers by screening an ester structure bearing not only an alkyl chain but also an aliphatic chain, including a heteroatomic moiety. In this study, OPs exhibiting various oxyethylene derivatives were synthesized, and their polymerization behavior was studied in detail using a [Rh(nbd)Cl]_2_ catalyst under several different conditions, such as the substituent effect, solvent, and reaction time. Interestingly, several of the synthesized POPs delivered excellent yields, and exhibited high molecular weights in methanol and *N*,*N*-dimethylformamide at 40 °C, compared with those of the PAPs. Furthermore, the monomer structure and intermediate conformation of the Rh complex were optimized by using DFT methods (B3LYP/6-31G** and B3LYP/LANL2DZ), and the polymerization mechanism was also proposed.

## 2. Experimental

### 2.1. Measurements

Number and weight average molecular weights (*M*_n_ and *M*_w_) of polymers were measured using JASCO GPC 900-1 equipped with two Shodex K-806L columns and an RI detector. CHCl_3_ was used as an eluent at 40 °C and poly(styrene) standards (*M*_n_ = 800−1,090,000) were employed for calibration. ^1^H NMR(500 MHz) and ^13^C NMR(125 MHz) spectra were measured on a JEOL JNM-ECA500 using chloroform-*d* (CDCl_3_) at 30 °C (Figures S1 and S2).

### 2.2. Materials

The chloroform (CHCl_3_), tetrahydrofuran (THF), toluene (Tol), acetone (Ace), ethyl acetate (EtOAc), acetonitrile (MeCN), *N*,*N*-dimethylformamide (DMF), dimethyl sulfoxide (DMSO), ethanol (EtOH), *iso*-propanol (IPA), Diethylene Glycol Monoethyl Ether Acetate (DEGMA), Diethylene Glycol Monomethyl Ether (DEGME) and water were used as solvent of a special grade. The oxyethyl propiolates (OP)s and *n*-butyl propiolate (BP(1)) were prepared with propiolic acid and corresponding alcohols in the presence of toluene sulfonic acid following a literature methods (Schemes S1 and S2) [42,43,44]. The syntheses of methoxyethyl propiolate (OP(1)) as the typical procedure were shown below.

#### Synthesis of 2-Methoxyethyl propiolate, OP(1)

A mixture of 80 mL of toluene, 9.13 g (0.12 mol) of 2-methoxy ethanol (Junsei Chem. Co., Ltd., Tokyo, Japan), 5.60 g (0.08 mol) of propiolic acid (Sigma-Aldrich Japan, Tokyo, Japan) and 1.52 g (8.0 mmol) of *p*-toluenesulfonic acid (Tokyo Chem. Ind., Tokyo, Japan) was refluxed for 8 h in a Dean-Stark apparatus. The resulting mixture was washed with an aqueous solution of saturated sodium hydrogen carbonate and distilled water. After the organic layer had been dried over anhydrous sodium sulfate, the solvent was removed with evaporation. The crude product was purified with silica-gel column chromatography (eluent: *n*-Hexane/ethyl acetate) to give a colorless oil OP(1), producing 6.87 g in a 67% yield. ^1^H NMR (500 MHz, CDCl_3_) δ: 4.35 (m, 2H, OCH_2_CH_2_), 3.64 (m, 2H, CH_2_CH_2_O), 3.40 (s, 3H, CH_3_), 2.90 (s, 1H, HC≡C), ^13^C NMR (ppm): δ 152.5, 75.2, 74.3, 69.7, 65.0, 58.9.

### 2.3. Polymerization

**Scheme 1:**
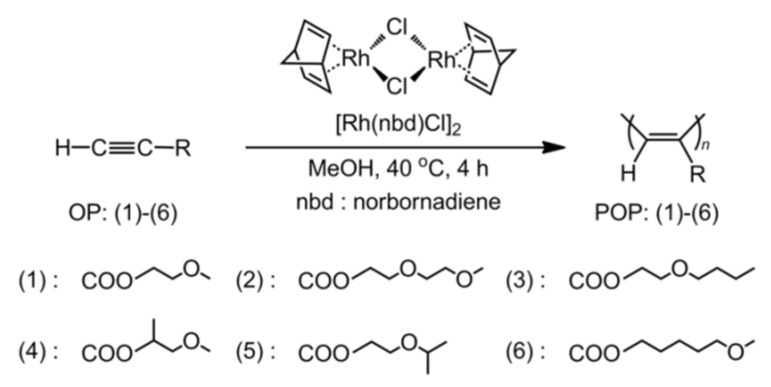
Synthesis of poly(oxyethyl propiolate)s using a [Rh(nbd)Cl]_2_ catalyst in MeOH.

Poly(oxyethyl propiolate)s (POPs) and poly(*n*-butyl propiolate) (PBP(1)) were obtained upon corresponding monomers using a catalyst, [Rh(nbd)Cl]_2_ (Wako Pure Chem. Ind., Osaka, Japan), as shown in Scheme 1 and Scheme S2. In a typical procedure, the syntheses of poly(methoxyethyl propiolate) (POP(1)) was shown below.

#### Synthesis of Poly(2-Methoxyethyl propiolate), POP(1)

1.0 g (7.8 mmol) of the monomer and a calculated quantity of the catalyst, 36 mg (7.8 × 10^−2^ mmol), were dissolved in MeOH (3.9 mL). The mixture was added to a sample tube and was stirred for 4 h at 40 °C. The resulting solution was poured into excess MeOH under stirring. The resulting polymer as fiber was washed with MeOH and dried under dynamic vacuum, ca. 10^−2^ Torr, for 12 h at room temperature. ^1^H NMR (500 MHz, CDCl_3_) δ: 6.90 (br., 1H, HC=C), δ 4.11, 3.98 (br., 2H, COOCH_2_), 3.56 (br., 2H, CH_2_O), 3.38 (br., 3H, CH_3_), ^13^C NMR (ppm): δ 163.8, 135.9, 127.9, 69.8, 58.9.

### 2.4. Computation

All calculations were performed with SPARTAN’14 program according to those in references [18,19,45,46]. The optimized structures of the monomers were obtained by the density functional theory (DFT) method using the B3YLP functional, which includes Becke’s three-parameter-exchange functionals, and the nonlocal Lee, Yang, and Parr correlation functional and the basis set 6-31G**. The Rh complexes were also optimized by the DFT method with the B3YLP functional and the basis set LANL2DZ.

## 3. Results and Discussion

### 3.1. Polymerization Behavior of OPs in MeOH

The polymerization of OP monomers using the [Rh(nbd)Cl]_2_ catalyst in MeOH for 4 h at 40 °C is shown in Table 1, including yield, *M*_n_, *M*_w_/*M*_n_, and *cis*%. The POPs that exhibited the normal oxyethylene group in the side-chain, e.g., POP(1), POP(2), and POP(3), delivered high yields (>70%) independent of the alkyl-chain length or the unit number of the oxyethylene moiety. The synthesis of POP(4), however, which had a branched ester moiety in its side-chain, achieved a yield 42% lower than POP(1), similar to the poly(2-alkyl propiolate)s [35,36,37,38,39,40,41,42,43,44]. POP(5), which exhibited a branched alkoxy group on its terminal in the side-chain, had a moderate polymerization yield of 58%. Moreover, the long methylene spacer decreased the polymer yield, compared to the POPs with normal oxyethylene groups in their side-chains. This is shown by the fact that the synthesis of POP(6), which had five methylene units, also delivered a moderate yield of 53%. These results indicate that POPs with oxyethyl ester moiety and normal alkoxy units (–COO–CH_2_–CH_2_–O–R, R = C_n_H_2n+1_) exhibited a high yield, compared with PAPs that had the aliphatic ester moiety [35,36,37,38,39,40,41,42,43,44]. Furthermore, the molecular weight (*M*_n_) of the POPs was relatively high, which exhibited approximately between 70,000 and 350,000, whereas the aliphatic PAP synthesized under the same conditions had a *M*_n_ of <70,000 [35,36,37,38,39,40,41,42,43,44].

### 3.2. Polymerization Behavior of OP(2) in Various Solvents

The solvent effect on the polymerization behavior of OP(2) was studied for various solvents, because OP(2) was polymerized homogeneously not only in organic solvent but also in water, except for the aliphatic solvent and the normal ether. As shown in Table 2, the polymerization of OP(2) exhibited moderate yields of >50% in EtOH, MeCN, DMF, and water. In particular, DMF and MeOH returned high yields. POP(2) was only slightly synthesized in IPA, THF, and acetone however, though the polymerization of OP(2) barely proceeded in EtOAc, toluene, and CHCl_3_. As for the previously described Rh catalyst system, the polymerization of OPs was also performed using a monomeric Rh complex as the catalytic active species. This was generated via the monomerization of [Rh(nbd)Cl]_2_ in a protic and/or electron-donating solvent such as alcohol, amide, or water [13,14,15,16,17,18,19,20,21,22]. The *M*_n_ of the polymers obtained using these various solvents were relatively high (~100,000–300,000). On the other hand, the polymerization of propiolate derivatives is explosively accelerated in the presence of a cocatalyst such as trimethylamine and triphenylphosphine accompanied with the dissociation of the Rh dimer complex in toluene. However, this reaction is too violent to control the stereoregular structure and/or molecular weight of the obtained polymers [22]. Moreover, we suppose that THF works as not only the dissociator of Rh dimer complex but also the accepter of hydrogen bonding between the hydrogen of acetylene moiety, because the acidity of propiolate derivatives is stronger than that of phenyl acetylene derivatives. Therefore, THF solvent was inefficient for the polymerization of OP(2).

These polymerization results suggest that the oxyethylene moiety promoted coordination between the monomer and the Rh complex catalyst, compared with that of the aliphatic APs. Furthermore, this strong coordination was able to accelerate the insertion of the monomer between the growing end of the polymer and the Rh complex catalyst, based on the polymerization mechanism of the coordination–insertion system. Therefore, the polymerization of OP(2) was also performed using both DEGMA and DEGME, which both have the same oxyethylen unit as OP(2), to clarify the coordination effect of the oxyethylene unit on the Rh complex catalyst (Table 2). However, POP(2) was barely obtained using DEGMA, and the polymerization of OP(2) also achieved only a low yield using DEGME. This result indicated that the oxyethylene unit did not activate the Rh complex catalyst during polymerization, but that the Rh complex catalyst could readily accept the monomer, due to the strong coordination between the two.

### 3.3. Polymerization Behavior of OP(1) and BP(1) Dependent on Reaction Time When Using MeOH

**Figure 1:**
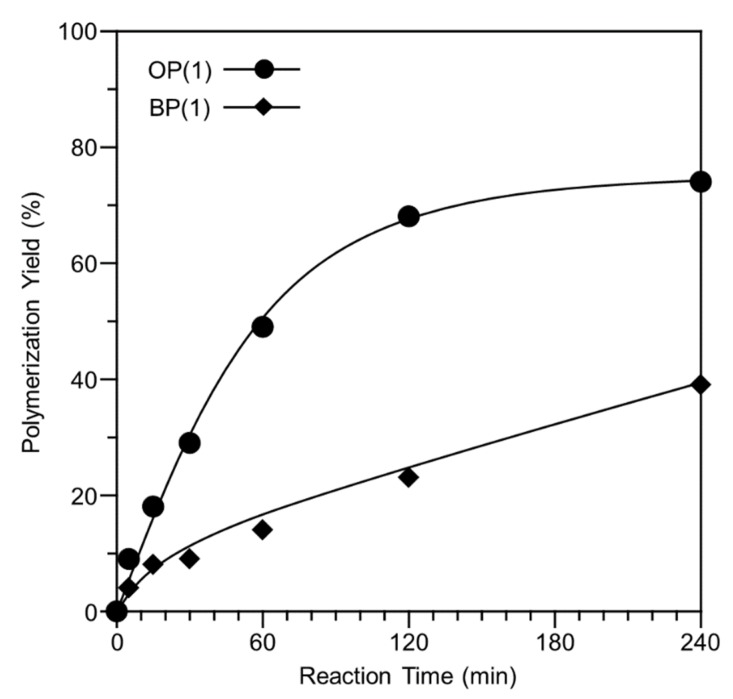
Effect of the reaction time on yield in the polymerization of OP(1) and BP(1) with [Rh(nbd)Cl]_2_ in MeOH at 40 °C ([M]_0_ = 2.0 M, [M]_0_/[cat.] = 100).

The polymerization of OP(1) and BP(1) was carried out using MeOH for various reaction times, in order to clarify the dependence of the polymerization reactivity on the monomer structure (Figure 1). The polymerization of both monomers proceeded rapidly for the first 15 min, although PBP(1) achieved only half of the yield achieved by POP(1), even at this initial step. However, the polymerization rate of BP(1) appreciably decreased after 30 min. Although the yield of PBP(1) was still approximately half of that of POP(1) after 240 min, the high yield of PBP(1) was expected under this long reaction time, because the polymerization yield of PBP(1) exhibited linear growth after 30 min. In fact, the polymerization of BP(1) has previously been reported to give a 44% yield after 6 h, and a 61% yield after 24 h [35,36,37,38,39,40,41,42,43,44]. The polymerization rate of OP(1) was efficiently accelerated, however, and had almost reached saturation after 240 min. The yield of OP(1) was 74% after 240 min already higher than that of BP(1) after 24 h. Therefore, the monomer structure of the OPs resulted in a higher yield and molecular weight for the obtained polymer using the conventional Rh catalyst system. Furthermore, the polymerization of BP(1) was carried out in MeOH with addition of DEGME as a cocatalyst ([M]_0_/[cat.] = 100, [cocat.]/[cat.] = 100, [M]_0_/[cocat] = 1). However, the polymerization yield of BP(1) somewhat decreased compared to that without a cocatalyst. This result also indicates that the solvent and/or cocatalyst are ineffective for the activation of Rh complex catalyst.

### 3.4. Relevance of Monomer Structure and Polymerization Behavior Based on DFT Calculation

**Figure 2:**
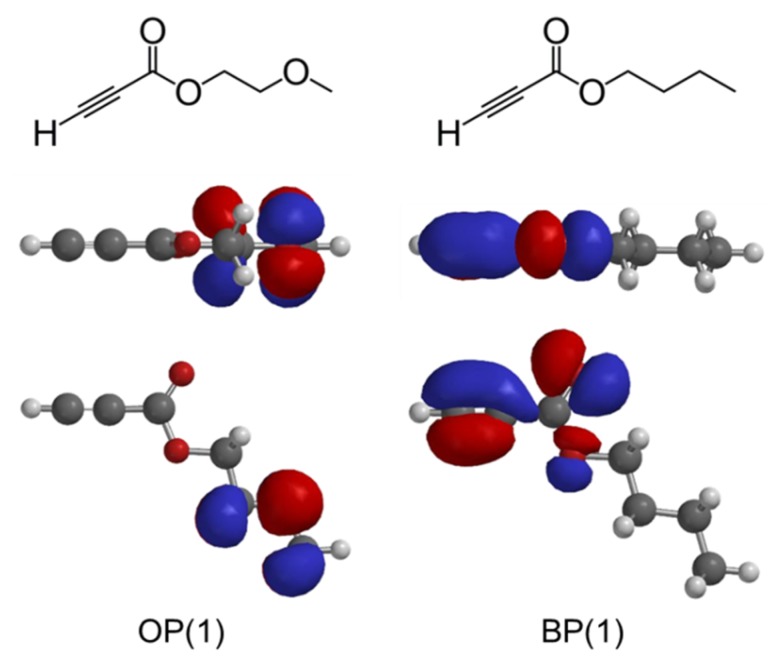
HOMO of OP(1) and BP(1) calculated using the DFT method (B3LYP/6-31G**).

DFT (B3LYP/6-31G**) calculations of the OP monomers were carried out to gain insight into the active moiety of the Rh complex catalyst. Figure 2 shows the highest occupied molecular orbital (HOMO) of OP(1) and BP(1) as estimated using DFT calculations. This orbital map strongly supports the interpretation that the coordination between the oxyethylene moiety and the Rh complex catalyst was able to occur readily because of the localized electron on the ether moiety of the oxyethylene unit in the HOMO of OP(1), although HOMO of BP(1) exhibited the spread electron on the acetylene and ester moieties. Moreover, the other OP monomers were also revealed to exhibit the same HOMO state as OP(1), independent of their oxyethylene structures, by the DMT calculations (Figure S3).

**Figure 3:**
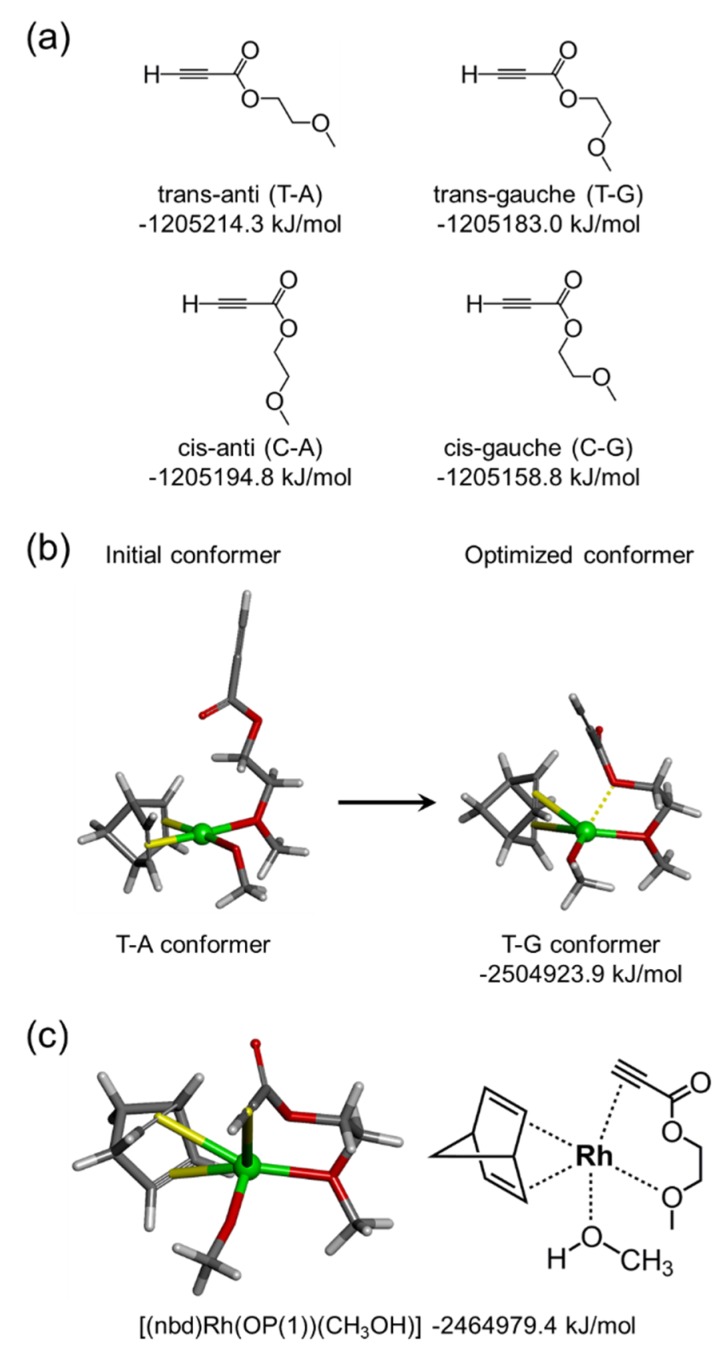
(**a**) Possible conformers of OP(1) and their energy as estimated by the DFT method (B3LYP/6-31G**). (**b**) The initial and optimized conformers of [(nbd)Rh(OP(1))(CH_3_OH)], calculated using the DFT method (B3LYP/LANL2DZ). (**c**) The intermediate conformer proposed by the DFT method (B3LYP/LANL2DZ).

Therefore, the coordination complex between OP(1) and the Rh complex catalyst was optimized using the DFT (B3LYP/LANL2DZ) calculation. Although OP(1) had four conformations, T-A conformation was used to optimize the coordination complex as the suitable initial structure of the monomer, because the optimized energy for T-A was the lowest of all conformations (Figure 3a). Here, the initial conformer of the Rh catalyst, which was tetracoordinated by OP(1) of the T-A conformation, nbd, and by using CH_3_OH as the solvent ligand, was optimized using the DFT method. After full geometry optimization, the conformation of the optimized OP(1) moiety changed from T-A to T-G, and the acetylene moiety was close to the rhodium (Figure 3b). This conformation change happened due to the weak interaction between the free ester oxygen and the rhodium having the coordinated ether oxygen, such as the chelate complex. Furthermore, the Rh conformer, [(nbd)Rh(OP(1))(CH_3_OH)], which showed coordination between the acetylene moiety and the rhodium, was also optimized from the T-G conformer. The optimized Rh conformer exhibited conformation including vertical coordination between the acetylene moiety and the rhodium (Figure 3c). Therefore, T-G conformation of the coordinated monomer helped the acetylene moiety and the rhodium to bond. These results of the DFT calculations indicate that the polymerization of the OP monomers was appreciably accelerated, compared to the AP monomers, due to the combination of the oxyethylene moiety and the Rh complex catalyst.

### 3.5. Proposal Polymerization Mechanism of OP(1) Using the Rh Complex Catalyst in MeOH

**Scheme 2:**
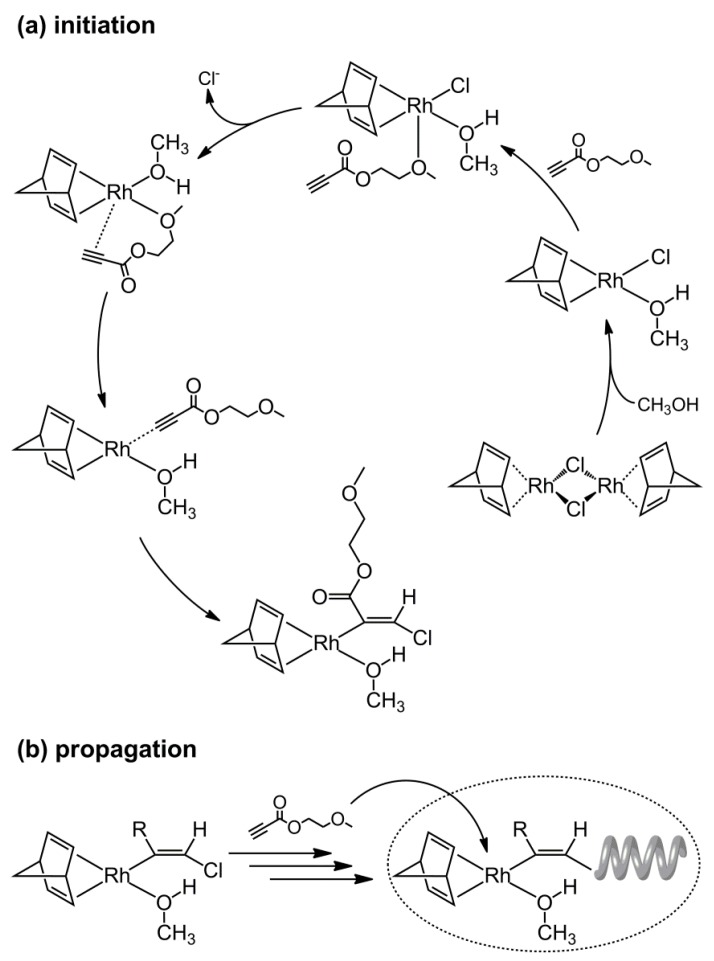
Possible and specious polymerization mechanism of OP(1) using [Rh(nbd)Cl]_2_ in MeOH, (**a**) initiation and (**b**) propagation steps.

On the basis of the DFT calculations, we supposed the specious polymerization mechanism. Possibly, a part of polymerization was initiated by forming the monomeric Rh complex as the active species, including the monomer moiety, whereas the major reaction of initiation step proceeded through the general pathway of propiolate and phenylacetylene derivatives (Scheme 2) [13,14,15,16,17,18,19,20,21,22]. Firstly, the monomeric Rh complex was generated via the coordination of methanol into the rhodium, the tetracoordinated Rh complex was then able to catch the monomer of the T-A conformation due to coordination between the ether oxygen and the rhodium. Secondly, the conformation of the oxyethylene moiety changed from T-A to T-G, together with the elimination of the chloride anion. Thirdly, the coordination of the oxyethylene moiety was released, and the acetylene moiety bonded with the rhodium (Scheme 2a). After this initiation pathway, the next monomer was then inserted between the initial monomer and the Rh complex catalyst. The propagation step also proceeded through the general pathway of mono-substituted acetylenes. In this step, the propagation is possibly accelerated due to the coordination between the oxyethylene moiety of monomers and Rh propagation species. (Scheme 2b).

## 4. Conclusions

The polymerization of OP was performed using the [Rh(nbd)Cl]_2_ catalyst in MeOH for 4 h at 40 °C. The POPs that exhibited the normal oxyethylene group in their side-chain delivered high yields and high molecular weights, independent of their alkyl-chain length and the unit number of their oxyethylene moiety, compared with the PAP derivatives. Furthermore, OPs were also synthesized at yields of ~50–70% yield, with molecular weights of 100,000–300,000, using protic and/or electron-donating solvents such as EtOH, MeCN, DMF, and water. The polymerization behavior was studied in detail using DMT calculations of the monomer and Rh complex conformations. After full geometry optimization, the Rh conformer exhibited conformation coordinating between the oxyethylene moiety (of gauche form) and the acetylene moiety as the monomer ligand. These results reveal that the polymerization of the OP monomers was appreciably accelerated, compared with the AP monomers, due to the combination of the monomer having the oxyethylene moiety, and the use of the Rh complex catalyst.

## Figures and Tables

**Table 1:** Synthesis of poly(oxyethy propiolate)s using a [Rh(nbd)Cl]_2_ catalyst in MeOH ^1^.

Polymer	Yield ^2^	*M*_n_^3^	*M*_w_/*M*_n_^3^	*Cis* ^4^
	(%)	(×10^−5^)		(%)
POP(1)	74	1.3	3.1	84
POP(2)	73	2.1	6.8	82
POP(3)	71	0.9	4.4	83
POP(4)	42	0.7	2.5	85
POP(5)	58	3.5	3.7	84
POP(6)	53	0.9	3.5	83

^1^ [M]_0_ = 2.0 mol/L, [M]_0_/[cat.] = 100. ^2^ Insoluble fraction in methanol or *n*-hexane. ^3^ Estimated by GPC analysis (PSt, CHCl_3_). ^4^ Determined by ^1^H NMR analysis (CDCl_3_).

**Table 2:** Synthesis of POP(2) using a [Rh(nbd)Cl]_2_ catalyst in various solvents ^1^.

Solvent	Yield ^2^	*M*_n_^3^	*M*_w_/*M*_n_^3^	*Cis* ^4^
	(%)	(×10^−5^)		(%)
EtOH	56	2.37	7.30	73
IPA	20	1.31	17.5	71
THF	8	0.02	1.39	53
EtOAc	trace	-	-	-
MeCN	52	2.84	2.65	63
DMF	71	1.85	11.2	78
Water	51	2.17	7.60	60
Toluene	trace	-	-	-
Acetone	12	0.81	3.35	49
CHCl_3_	trace	-	-	-
DEGMA	trace	-	-	-
DEGME	16	0.77	2.33	58

^1^ [M]_0_ = 2.0 mol/L, [M]_0_/[cat.] = 100. ^2^ Insoluble fraction in *n*-hexane. ^3^ Estimated by GPC analysis (PSt, CHCl_3_). ^4^ Determined by ^1^H NMR analysis (CDCl_3_).

## Supplementary Materials

The following are available online at http://www.mdpi.com/2073-4360/11/1/93/s1, Scheme S1: ( a) Synthesis of monomers, OP(1)–(6), with esterification in the present of the acid catalyst. (b) Synthesis of 5-Methoxy-1-pentanol with valerolactone as starting material, which prepared through methyl 5-methoxypentanoate, Scheme S2: Synthesis of *n*BuP with esterification in the present of the acid catalyst and polymerization of BP(1) with [Rh(nbd)Cl]_2_ catalyst in MeOH, Figure S1: ^1^H NMR spectra of POPs observed in CDCl_3_ at room temperature, Figure S2: ^13^C NMR spectra of POPs observed in CDCl_3_ at room temperature, Figure S3: HOMO of OPs calculated using the DFT method (B3LYP/6-31G**).
