# Supplementary material for: Proposed Mechanism for the High-Yield Polymerization of Oxyethyl Propiolates with Rh Complex Catalyst Using the Density Functional Theory Method
Source: Polymers (Basel). 2019 Jan 8;11(1):93. doi: 10.3390/polym11010093 (PMC6402018; doi:10.3390/polym11010093)
Supplement: Supplementary file 1 [file polymers-11-00093-s001.pdf]

Supporting Information for

Proposed Mechanism for the High-Yield Polymerization of Oxyethyl Propiolates with Rh Complex Catalyst using the Density Functional Theory Method

Yoshiaki Yoshida[1‡], Yasuteru Mawatari[1,2*], and Masayoshi Tabata[3,4*]

[1] Graduate School of Engineering, Muroran Institute of Technology, 27-1 Mizumoto-cho, Muroran, Hokkaido 050-8585, Japan

[2] *Research Center for Environmentally Friendly Materials Muroran Institute of Technology, 27-1 Mizumoto-cho, Muroran, Hokkaido 050-8585, Japan*

[3] *Center of Environmental Science and Disaster Mitigation for Advanced Research, Muroran Institute of Technology, 27-1 Mizumoto-cho, Muroran, Hokkaido 050-8585, Japan*

[4] *Faculty of Science and Technology, Department of Applied Chemistry and Bioscience, Chitose Institute of Science and Technology, Bibi 65-758, Hokkaido Chitose 066-8655, Japan*

*Correspondence: mawatari@mmm.muroran-it.ac.jp (Y.M.); tabata@mmm.muroran-it.ac.jp (M.T.); Tel.: +81-143-46-5964 (Y.M.); +81-143-46-5963 (M.T.)

EXPERIMENTAL

Synthesis of monomers

The monomers were prepared with 5.60 g (0.08 mol) of propiolic acid, corresponding alcohol (0.12 mol) and 1.52 g (8.0 mmol) of *p*-toluenesulfonic acid was refluxed for 8 h in a Dean-Stark apparatus according to the procedure of the main manuscript (**Scheme S1a**). The 5-methoxy-1-pentanol was prepared following a literature methods as mentioned below (**Scheme S1b**) [11].

2-(2-methoxyethoxy)ethyl propiolate, OP(2): The OP(2) was prepared with 14.4 g (0.12 mol) of diethylene glycol monomethyl ether (Junsei Chem. Co., Ltd., Tokyo Japan). The crude product was purified with distillation (96 ºC/3 mmHg) to give a colourless liquid OP(2), producing 12.0 g in a 87% yield. ^1H NMR (500 MHz, $CDCl_3$) δ: 4.36 (m, 2H, $COOC\underline{H}_2CH_2$), 3.75 (m, 2H, $COOCH_2C\underline{H}_2O$), 3.66 (m, 2H, $OCH_2C\underline{H}_2OCH_3$), 3.56 (m, 2H, $OC\underline{H}_2CH_2OCH_3$), 3.40 (s, 3H, $C\underline{H}_3$), 2.90 (s, 1H, $\underline{H}C{\equiv}C$), ^{13}C NMR (ppm): δ 152.6, 75.1, 74.4, 71.8, 70.5, 68.5, 65.1, 59.0.

2-Butoxyethyl propiolate, OP(3): The OP(3) was prepared with 14.2 g (0.12 mol) of ethylene glycol monobutyl ether (Tokyo Chem. Ind., Tokyo Japan). The crude product was purified with distillation (80 ºC/6 mmHg) to give a colourless liquid OP(3), producing 12.1 g in a 89% yield. ^1H NMR (500 MHz, $CDCl_3$) δ: 4.34 (m, 2H, $COOC\underline{H}_2$), 3.66 (m, 2H, $COOCH_2C\underline{H}_2O$), 3.48 (t, J = 6.6 Hz, 2H, $CH_2OC\underline{H}_2$), 2.90 (s, 1H, $\underline{H}C{\equiv}C$), 1.57 (m, 2H, $OCH_2C\underline{H}_2CH_2$), 1.37 (m, 2H, $CH_2C\underline{H}_2CH_3$), 0.92 (t, J = 7.3 Hz, 3H, $C\underline{H}_3$), ^{13}C NMR (ppm): δ 152.7, 74.61, 74.56, 72.3, 66.1, 58.4, 29.0, 28.0, 22.4.

1-methoxypropan-2-yl propiolate, OP(4): The OP(4) was prepared with 10.8 g (0.12 mol) of 1-methoxy-2-propanol (Tokyo Chem. Ind., Tokyo Japan). The crude product was purified with distillation (73 ºC/18 mmHg) to give a colourless liquid OP(4), producing 9.52 g in a 83% yield. ^1H NMR (500 MHz, $CDCl_3$) δ: 5.19 (m, 1H, $COOC\underline{H}$), 3.46 (m, 2H, $CHC\underline{H}_2O$), 3.38 (s, 3H, $OC\underline{H}_3$), 2.88 (s, 1H, $\underline{H}C{\equiv}C$), 1.30 (d, J = 6.4 Hz, 3H, $CHC\underline{H}_3$), ^{13}C NMR (ppm): δ 152.1, 74.77, 74.70, 74.39, 71.6, 59.0, 16.2.

2-Isopropoxyethyl propiolate, OP(5): The OP(5) was prepared with 12.5 g (0.12 mol) of ethylene glycol monoisopropyl ether (Tokyo Chem. Ind., Tokyo Japan). The crude product was purified with distillation (68 ºC/4 mmHg) to give a colourless liquid OP(5), producing 11.6 g in a 93% yield. ^1H NMR (500 MHz, $CDCl_3$) δ: 4.32 (m, 1H, $COOC\underline{H}_2$), 3.66 (m, 2H, $CHC\underline{H}_2O$), 3.63 (m, 1H, $OC\underline{H}$), 2.90 (s, 1H, $\underline{H}C{\equiv}C$), 1.18 (d, J = 6.1 Hz, 6H, $(C\underline{H}_3)_2$), ^{13}C NMR (ppm): δ 152.6, 75.1, 74.5, 72.0, 65.5, 65.2, 21.9.

Methyl 5-methoxypentanoate: 0.5 mL (9.0 mmol) of Sulfuric acid was added to a stirred solution of 25.0 g (0.25 mol) of valerolactone (Tokyo Chem. Ind., Tokyo Japan), 50.0 g (0.47 mol) of trimethyl orthoformate (Tokyo Chem. Ind., Tokyo Japan) and 100 mL of MeOH. The mixture was heated at

50 °C for 24 h. The resulting mixture was washed with an aqueous solution of saturated sodium hydrogen carbonate, distilled water and brine. Distillation (98 ºC/50 mmHg) of the crude product provided 31.8 g (87%) of methyl 5-methoxypentanoate as colourless liquid. ^{1}H NMR (500 MHz, $CDCl_3$) δ: 3.67 (s, 1H, $COOC\underline{H}_3$), 3.38 (t, J = 6.4 Hz, 2H, $C\underline{H}_2OCH_3$), 3.33 (s, 1H, $OC\underline{H}_3$), 2.34 (t, J = 7.2 Hz, 2H, $OCC\underline{H}_2$), 1.70 (m, 2H, $OCCH_2C\underline{H}_2$), 1.61 (m, 2H, $C\underline{H}_2CH_2O$), ^{13}C NMR (ppm): δ 152.6, 74.66, 74.56, 68.6, 63.4, 58.6, 28.5.

5-methoxy-1-pentanol: A solution of 40 ml of anhydrous Et_2O and 30.3 g of methyl 5-methoxypentanoate was slowly added dropwise into a stirred mixture of 380 ml of anhydrous Et_2O and 11.0 g of lithium aluminium hydride (kanto chem. Co., inc., Tokyo Japan) in ice bath. The ice bath was removed and the reaction mixture was stirred for 24 h at room temperature. The resulting mixture was cooled with ice bath and a 11 ml of distilled water was added dropwise into the reaction mixture to keep stirring. The resulting suspension of a gray slurry was quenched with 11 ml of 15% aqueous sodium hydroxide and 33 ml of distilled water. The gray slurry was separated with filtration with 3 times 150 ml portions of EtOAc and the filtrate was washed with distilled water and brine. After the organic layer had been dried over anhydrous sodium sulfate, the solvent was removed with evaporation. The crude product was purified with distillation (62 ºC/4 mmHg) to give a colourless liquid of 5-methoxy-1-pentanol, producing 20.5 g in a 83% yield. ^{1}H NMR (500 MHz, $CDCl_3$) δ: 3.65 (br., 1H, $C\underline{H}_2OH$), 3.39 (t, J = 6.4 Hz, 2H, $C\underline{H}_2OCH_3$), 3.34 (s, 1H, $OC\underline{H}_3$), 1.60 (m, 4H, $C\underline{H}_2CH_2C\underline{H}_2$), 1.56 (m, 1H, $O\underline{H}$), 1.43 (m, 2H, $CH_2C\underline{H}_2CH_2$), ^{13}C NMR (ppm): δ 72.7, 62.8, 58.6, 32.5, 29.3, 22.3.

5-methoxypentyl propiolate, OP(6): The OP(6) was prepared with 11.8 g (0.12 mol) of prepared 5-methoxy-1-pentanol. The crude product was purified with distillation (82 ºC/4 mmHg) to give a colourless liquid of OP(6), producing 12.3 g in a 90% yield. ^{1}H NMR (500 MHz, $CDCl_3$) δ: 4.20 (t, J = 6.3 Hz, 2H, $COOC\underline{H}_2$), 3.38 (t, J = 6.3 Hz, 2H, $C\underline{H}_2OCH_3$), 3.33 (s, 1H, $OC\underline{H}_3$), 2.88 (s, 1H, $\underline{H}C\equiv C$), 1.71 (m, 2H, $COOCH_2C\underline{H}_2$), 1.61 (m, 2H, $CH_2C\underline{H}_2OC\underline{H}_3$), 1.45 (m, 2H, $CH_2C\underline{H}_2CH_2$), ^{13}C NMR (ppm): δ 152.3, 75.1, 74.4, 71.1, 67.8, 65.2, 31.5, 19.1, 13.8.

Polymerization

Poly(2-(2-methoxyethoxy)ethyl propiolate), POP(2): 1.0 g (5.8 mmol) of the monomer and a calculated quantity of the catalyst, 27 mg (5.8×10^{-2} mmol), were dissolved in MeOH (2.9 ml). The resulting solution was added 8 mL of $CHCl_3$ and poured into excess *n*-hexane under stirring. The resulting polymer as fiber was washed with *n*-hexane, producing 0.73 g after drying under dynamic vacuum. ^{1}H NMR (500 MHz, $CDCl_3$) δ: 6.80 (br., 1H, $\underline{H}C{=}C$), δ 4.14, 3.98 (br., 2H, $COOC\underline{H}_2$), 3.65 (br., 4H, $(C\underline{H}_2)_2OCH_3$), 3.54 (br., 2H, $COOCH_2C\underline{H}_2$), 3.37 (br, 3H, $C\underline{H}_3$), ^{13}C NMR (ppm): δ 163.7, 135.5, 128.0, 71.9, 70.6, 68.3, 64.3, 58.8.

Poly(2-Butoxyethyl propiolate), POP(3): 1.0 g (5.9 mmol) of the monomer and a calculated quantity of the catalyst, 27 mg (5.9 x 10^{-2} mmol), were dissolved in MeOH (2.9 ml). The resulting solution was poured into excess MeOH under stirring. The resulting polymer as powder was washed with MeOH, producing 0.71 g after drying under dynamic vacuum. ^{1}H NMR (500 MHz, $CDCl_3$) δ: 6.79 (br., 1H, $\underline{H}C{=}C$), δ 4.14, 3.92 (br., 2H, $COOC\underline{H}_2$), 3.57 (br., 2H, $COOCH_2C\underline{H}_2$), 3.47 (br., 2H, $OC\underline{H}_2$), 1.55 (br., 2H, $OCH_2C\underline{H}_2$), 1.37 (br., 2H, $C\underline{H}_2CH_3$), 0.93 (br, 3H, $C\underline{H}_3$), ^{13}C NMR (ppm): δ163.7, 135.7, 128.0, 71.1, 67.8, 63.9, 31.8, 19.2, 13.9.

Poly(1-methoxypropan-2-yl propiolate), POP(4): 1.0 g (7.0 mmol) of the monomer and a calculated quantity of the catalyst, 32 mg (7.0 x 10^{-2} mmol), were dissolved in MeOH (3.5 ml). The resulting solution was added 8 mL of $CHCl_3$ and poured into excess *n*-hexane under stirring. The resulting polymer as powder was washed with *n*-hexane, producing 0.42 g after drying under dynamic vacuum. ^{1}H NMR (500 MHz, $CDCl_3$) δ: 7.06 (br., 1H, $\underline{H}C{=}C$), δ 4.91 (br., 1H, $COOC\underline{H}$), 3.46 (br., 2H, $OCHC\underline{H}_2$), 3.33 (br., 3H, $OC\underline{H}_3$), 1.23 (br., 3H, $CHC\underline{H}_3$), ^{13}C NMR (ppm): δ 164.2, 137.5, 129.4, 74.4, 70.5, 59.0, 16.6.

Poly(2-Isopropoxyethyl propiolate), POP(5): 1.0 g (6.4 mmol) of the monomer and a calculated quantity of the catalyst, 30 mg (6.4 x 10^{-2} mmol), were dissolved in MeOH (3.2 ml). The resulting solution was added 8 mL of MeOH and poured into excess water under stirring. The resulting polymer as fiber was washed with water, producing 0.58 g after drying under dynamic vacuum. ^{1}H NMR (500 MHz, $CDCl_3$) δ: 6.80 (br., 1H, $\underline{H}C{=}C$), δ 4.12, 3.88 (br., 2H, $COOC\underline{H}_2$), 3.57 (br., 2H, $CH_2C\underline{H}_2O$), 3.62 (br., 1H, $C\underline{H}$), 1.16 (br., 6H, $(C\underline{H}_3)_2$), ^{13}C NMR (ppm): δ 163.6, 135.4, 128.0, 71.7, 65.0, 64.2, 22.0.

Poly(5-methoxypentyl propiolate), POP(6): 1.0 g (5.9 mmol) of the monomer and a calculated quantity of the catalyst, 27 mg (5.9 x 10^{-2} mmol), were dissolved in MeOH (2.9 ml). The resulting solution was added 8 mL of $CHCl_3$ and poured into excess *n*-hexane under stirring. The resulting polymer as powder was washed with *n*-hexane, producing 0.53 g after drying under dynamic vacuum. ^{1}H NMR (500 MHz, $CDCl_3$) δ: 6.76 (br., ^{1}H, $\underline{H}C{=}C$), δ 3.95, 3.72 (br., 2H, $COOC\underline{H}_2$), 3.38 (br., 2H, $C\underline{H}_2O$), 3.32 (br., 3H, $C\underline{H}_3$), 1.60 (br., 4H, $OCH_2(C\underline{H}_2)_2CH_2$), 1.38 (br., 2H, $C\underline{H}_2CH_2O$), ^{13}C NMR (ppm): δ 163.8, 135.3, 127.8, 72.5, 64.9, 58.4, 29.2, 27.9, 22.3.

Synthesis of n-Butyl propiolate and Poly(n-butyl propiolate) [11]

n-Butyl propiolate, BP(1): The BP(1) was prepared with 8.89 g (0.12 mol) of *n*-butanol (Tokyo Chem. Ind.). The crude product was purified with distillation (60 ºC/60 mmHg) to give a colourless liquid BP(1), producing 4.75 g in a 57% yield. ^{1}H NMR (500 MHz, $CDCl_3$) δ: 4.20 (t, J = 6.6 Hz, 2H, $COOC\underline{H}_2$), 2.92 (s, 1H, $\underline{H}C{\equiv}C$), 1.72-1.62 (m, 2H, $OCH_2C\underline{H}_2$), 1.48-1.34 (m, 2H, $CH_2C\underline{H}_2CH_3$), 0.95 (t, J = 7.3 Hz, 3H, $C\underline{H}_3$), ^{13}C NMR (ppm): δ 152.4, 74.5, 65.9, 30.2, 18.9, 13.5.

Poly(n-butyl propiolate), PBP(1): 1.0 g (7.9 mmol) of the monomer and a calculated quantity of the catalyst, 36 mg (7.9 x 10^{-2} mmol), were dissolved in MeOH (4.0 ml). The mixture was added to a sample tube and was stirred for 4 h at 40 ℃. The resulting solution was poured into excess MeOH under stirring. The resulting polymer was washed with MeOH and dried under dynamic vacuum, *ca*. 10^{-2} Torr, for 12 h at room temperature. The producing polymer as powder was obtained 0.39 g in a 39% yield. ^{1}H NMR (500 MHz, $CDCl_3$) δ: 6.81 (br., 1H, <u>H</u>C=C), δ 3.97, 3.74 (br., 2H, $COOC\underline{H}_2$), 1.58 (br., 2H, $OCH_2C\underline{H}_2$), 1.37 (br., 2H, $CH_2C\underline{H}_2CH_3$), 0.93 (br., 3H, $C\underline{H}_3$), ^{13}C NMR (ppm): δ 163.7, 135.1, 127.6, 64.7, 30.2, 19.1, 13.7

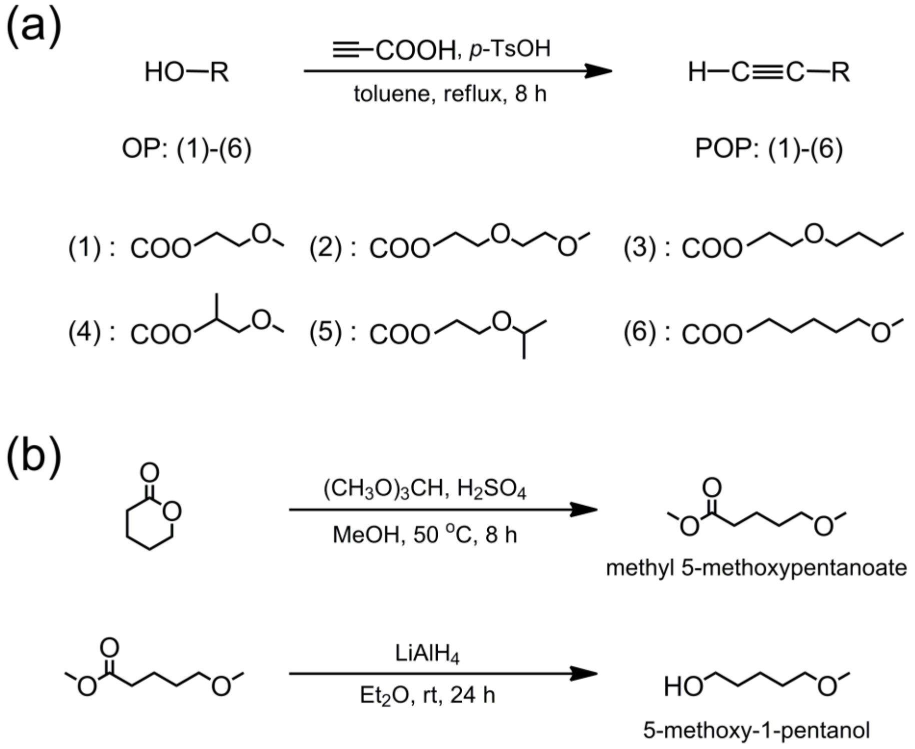

Scheme S1. (a) Synthesis of monomers, OP(1)-(6), with esterification in the present of the acid catalyst. (b) Synthesis of 5-Methoxy-1-pentanol with valerolactone as starting material, which prepared through methyl 5-methoxypentanoate.

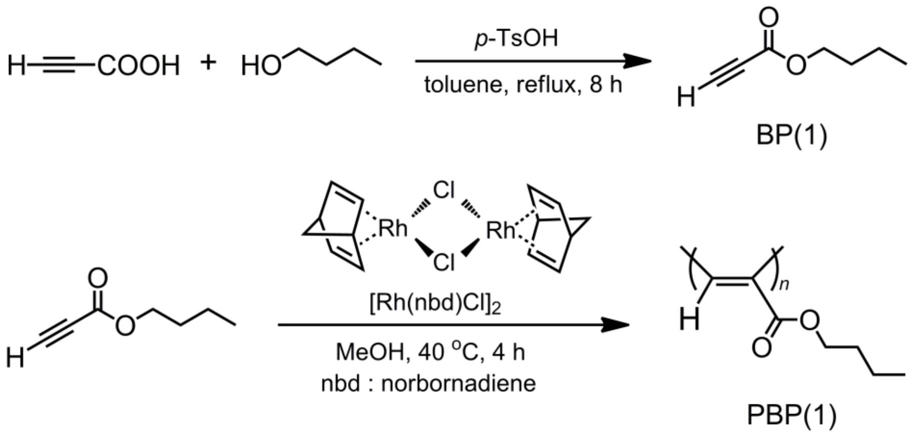

Scheme S2. Synthesis of *n*BuP with esterification in the present of the acid catalyst and polymerization of BP(1) with $[Rh(nbd)Cl]_2$ catalyst in MeOH.

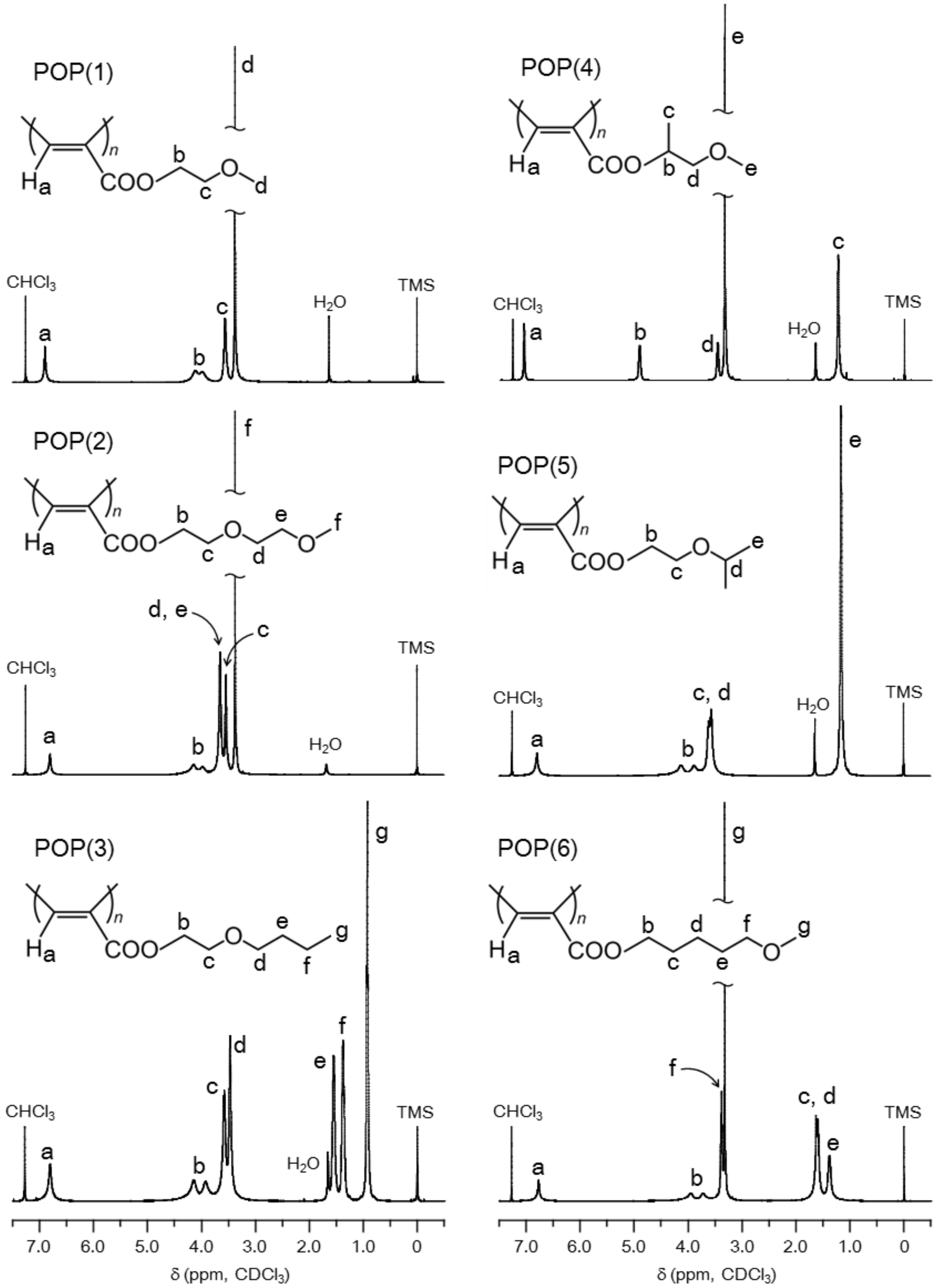

Figure S1. ^{1}H NMR spectra of POPs observed in $CDCl_3$ at room temperature.

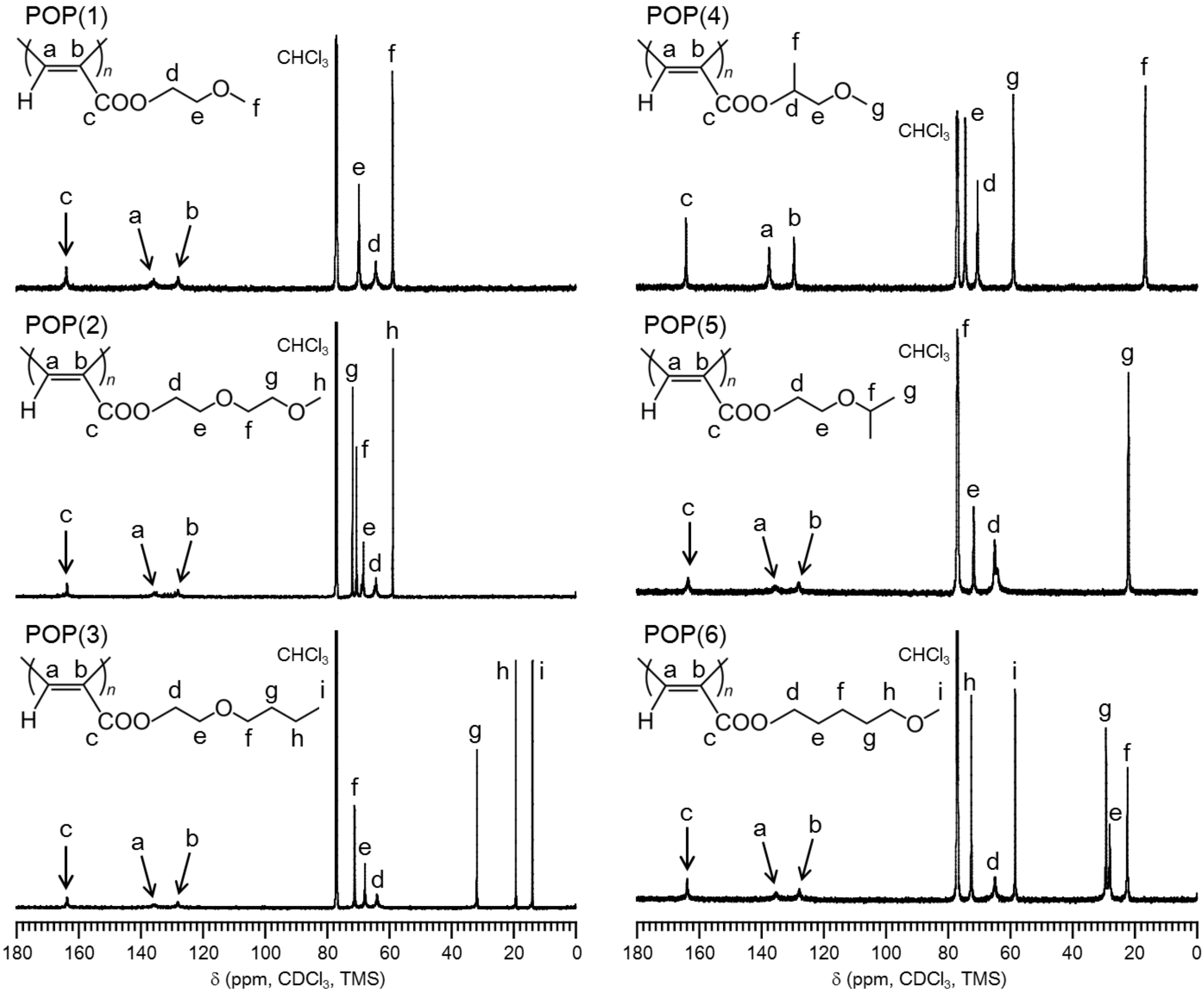

Figure S2. ^{13}C NMR spectra of POPs observed in $CDCl_3$ at room temperature.

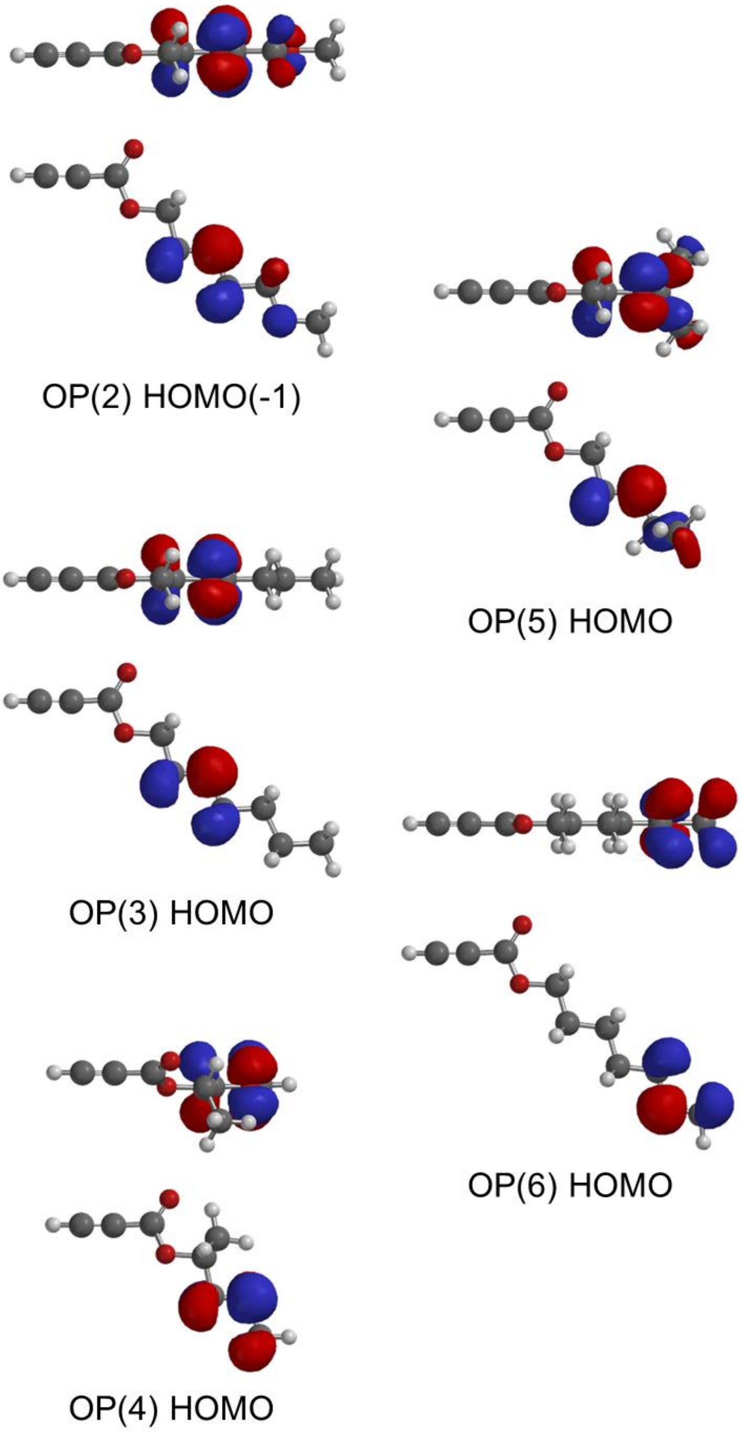

Figure S3. HOMO of OPs calculated using the DFT method (B3LYP/6-31G**).
